# Innate Immune Responses to Highly Pathogenic Coronaviruses and Other Significant Respiratory Viral Infections

**DOI:** 10.3389/fimmu.2020.01979

**Published:** 2020-08-18

**Authors:** Hanaa Ahmed-Hassan, Brianna Sisson, Rajni Kant Shukla, Yasasvi Wijewantha, Nicholas T. Funderburg, Zihai Li, Don Hayes, Thorsten Demberg, Namal P. M. Liyanage

**Affiliations:** ^1^Department of Microbial Infection and Immunity, College of Medicine, The Ohio State University, Columbus, OH, United States; ^2^Department of Zoonoses, Faculty of Veterinary Medicine, Cairo University, Giza, Egypt; ^3^Division of Medical Laboratory Science, School of Health and Rehabilitation Sciences, The Ohio State University, Columbus, OH, United States; ^4^The James Comprehensive Cancer Center, Pelotonia Institute for Immuno-Oncology, The Ohio State University, Columbus, OH, United States; ^5^Division of Pulmonary Medicine, Cincinnati Children's Hospital Medical Center, Cincinnati, OH, United States; ^6^Department of Pediatrics, University of Cincinnati College of Medicine, Cincinnati, OH, United States; ^7^Marker Therapeutics Inc., Houston, TX, United States; ^8^Department of Veterinary Biosciences, College of Veterinary Medicine, Ohio State University, Columbus, OH, United States; ^9^Infectious Diseases Institute, The Ohio State University, Columbus, OH, United States

**Keywords:** SARS-CoV-2, COVID-19, innate immune responses, Coronavirus (CoV), Coronavirus (2019-nCoV) outbreak

## Abstract

The new pandemic virus SARS-CoV-2 emerged in China and spread around the world in <3 months, infecting millions of people, and causing countries to shut down public life and businesses. Nearly all nations were unprepared for this pandemic with healthcare systems stretched to their limits due to the lack of an effective vaccine and treatment. Infection with SARS-CoV-2 can lead to Coronavirus disease 2019 (COVID-19). COVID-19 is respiratory disease that can result in a cytokine storm with stark differences in morbidity and mortality between younger and older patient populations. Details regarding mechanisms of viral entry via the respiratory system and immune system correlates of protection or pathogenesis have not been fully elucidated. Here, we provide an overview of the innate immune responses in the lung to the coronaviruses MERS-CoV, SARS-CoV, and SARS-CoV-2. This review provides insight into key innate immune mechanisms that will aid in the development of therapeutics and preventive vaccines for SARS-CoV-2 infection.

## Introduction

Severe Acute Respiratory Syndrome Coronavirus 2 (SARS-CoV-2) reportedly emerged at a live animal market in the Chinese city of Wuhan is currently causing a pandemic and negatively affecting global health (1–3). There are more than 11 million confirmed SARS-CoV-2 infections with an mortality rate that widely varies by country and region (4). Even in industrialized countries, SARS-CoV-2 led healthcare systems approach the brink of collapse by overwhelming their capacity and straining resources. Governments and local leaders ordered the shutdown of cities, regions, countries leading to massive disruptions in the local and global economy. Unlike previous Coronavirus (CoV) infections, the rapid global spread, high transmission rate, longer incubation time, and disease severity of SARS-CoV-2 requires a better understanding of the epidemiology and immunopathogenesis of this viral outbreak in order to learn from this experience and to manage future pandemics.

SARS-CoV-2 is a highly pathogenic CoV (5) (case-fatality rate of 3.6–3.8%) (4, 6) that is related to Severe Acute Respiratory Syndrome CoV (SARS-CoV) (case-fatality rate of 14–15%) and the Middle East Respiratory Syndrome CoV (MERS-CoV) (case-fatality rate of 34.4%), see also Table 1 (138, 139). SARS-CoV, SARS-CoV-2 and MERS-CoV target the lower respiratory system, causing respiratory illnesses, including severe pneumonia, acute lung injury (ALI) and acute respiratory distress syndrome (ARDS) (140, 141). SARS-CoV-2 infection results in higher hospitalization rates in the elderly (>65) and persons with pre-existing conditions including hypertension, diabetes and obesity compared to rates among younger populations without pre-existing conditions (Table 1) (142, 143). In addition to an age disparity, males with COVID-19 appear to have higher risk for worse outcomes and death (143, 144). Epidemiological research of the SARS and MERS infections also showed that males had a higher mortality rate than females (144–146).

**Table 1:** Comparison of Immune pathogenesis between highly pathogenic coronaviruses and other significant respiratory viral infections.

	**SARS-CoV**	**MERS-CoV**	**SARS-CoV-2**	**Influenza virus (IV)**	**Parainfluenza virus (PIV)**	**Respiratory syncytial virus (RSV)**	**Rhinovirus (RV)**
Receptor/s	ACE2 (7)	DPP4 (8)	ACE2 (9) DPP4 (10)	α-2,3 linkage and α-2,6 linkage (11)	α2,3-linked sialic acids (12)	CX3CR1 (13)	ICAM-1, LDLR, and CDHR3 (14)
Target cells	Multiple cell types in the lower respiratory tract were found to be infected, including type I alveolar epithelium, macrophages, and putative CD34^+^ Oct-4^+^ stem/progenitor cells in human lungs (15–17) Ciliated bronchial epithelial cells and type II pneumocytes (7, 18)	Un-ciliated bronchial epithelial cells and type II pneumocytes (19–21)	Infect mostly human type I and type II pneumocytes and alveolar macrophages (22) Respiratory, nasal, corneal and intestinal epithelial cells (23)	Club cells, ciliated cells, type I and type II alveolar cells (24)	Ciliated epithelial cells of the upper and lower respiratory tract (25)	The ciliated cells of the human airway epithelium are the main target, it also infects basal cells (26) and immune cells, such as Macrophages, B cells CD4^+^ and CD8^+^ T cells (27)	Upper and lower airways epithelial cell (28)
Mortality	11% (29)	34.4% (30)	3–4% (31)	<0.1% (31)	Unusual in developed countries. Preschool population in developing countries has considerable risk of HPIV-induced death. LRI causes 25 to 30% of the deaths in this age group and HPIV causes at least 10% of the LRI (32, 33)	Children <5 years—death uncommon, estimated at 100-500/year. Among US adults, an estimated 14,000 deaths/year (34)	–
Effected age	While younger individuals below 18 years of age experience mild-moderate clinical illness, elderly individuals exhibit worse outcomes after infection with SARS-CoV (35)	While younger individuals below 18 years of age experience mild-moderate clinical illness, elderly individuals exhibit worse outcomes after infection with MERS-CoV (36, 37)	Patients aged ≥ 60 years showed heavier clinical manifestations, greater severity and longer disease courses compared with those aged <60 years (38)	The influenza virus with highest sRIR was A(H1N1) for young children, B for older children, A(H1N1)pdm2009 for adults, and A(H3N2) for the elderly (39)	Persons of any age (40)	The highest burden of RSV was observed in young infants aged 3–5 months, whereas the burden was also high in those aged 12–20 months (41) and certain adult populations. These include the elderly, persons with cardiopulmonary diseases, and immunocompromised hosts (42)	RV was more frequently detected in younger children and infants than in older children (43)
R0—the reproduction number*	In the range of 2–4 (44)	*Saudi Arabia:* 0.45–0.98 (Only one study reported 1.9–3.9) *South Korea:* 2.5–8.1 and <1 in later period or with control intervention (45)	Between 2 and 2.5 (31)	Between 1.28 and 1.8 (46)	–	0.92–1.33 for RSV-A and 1.04–1.76 for RSV-B (47)	1.2–1.83 (48)
Incubation period	Mean, 5 days; range, 2 to 10 days (49)	5 to 7 days; range, 2 to 14 days (50)	Mean, 5 days; range, 2–14 days (51)	2 days; range, 1 to 4 days (52)	2–7 days (53)	4–6 days (54)	Mean, 1.9 days (52)
Serial interval time (the time between successive cases)	Mean, in Singapore 8.4 days (55)	12.6–14.6 days (45)	5–6 days (56)	3 days (31)	–	3.2 days (47)	–
Comorbidities	Diabetes, other comorbidities (chronic obstructive pulmonary disease, cancer, cardiac disease), and age of 60 years or older (57) acute renal impairment and proteinuria (58)	Diabetes mellitus, hypertension, ischemic heart disease, congestive heart failure, end-stage renal disease and chronic kidney disease (59)	>60 years and those with comorbid conditions, such as diabetes, hypertension and cardiovascular disease (CVD) (60–62)	Asthma; diabetes; heart, lung, and neurologic diseases; and pregnancy (63)	Immunocompromised and elderly adults (25)	Older adults (64) adults hospitalized with cardiopulmonary infections (65)	Asthma, chronic medical conditions, malignancies, or immunosuppression, (66–68)
**Immune responses**							
Macrophages	Non-productive infections (69)	Productive infections (69)	CD169^+^ macrophages could contribute to viral spread, excessive inflammation and activation-induced lymphocytic cell death during SARS-CoV-2 infection (70)	Non-productive infections more than 90% of resident AMs were lost in the first week after influenza, while the remaining cells had a necrotic phenotype. Result in significant morbidity through several pathways, including facilitation of secondary bacterial pneumonia (71)	Productive infections (72)	Productive infections (73) one of the foremost and only sources of IFN-I, contributing to the establishment of an antiviral state in neighboring cells (74)	Productive infections Rhinovirus replication in human macrophages causes activation and nuclear translocation of NF-κB, leading to TNF-α production (75)
Monocytes	SARS-CoV-infected human monocytes produce chemokines that attract the migration of neutrophils, macrophages, and activated T lymphocytes (76, 77)	MDMs were permissive for MERS-CoV (78)	Decreased (79)	Influenza infection markedly inhibit the monocyte chemotactic response and depress the phagocytosis (80)	Inefficient infection of Immature MDDCs and sub-optimal maturation (81)	Inefficient infection of Immature MDDCs and sub-optimal maturation (81)	Airway epithelial cells direct significant RV16 replication in monocytic cells via an ICAM1-dependent mechanism (82)
DC	SARS-CoV-infection was abortive in MDDCs (83)	Immature MDDCs were permissive for MERS-CoV infection, while mature MDDCs were not (78)	Activated dendritic cells increased (84)	IV was internalized by both myeloid DCs (mDCs) and plasmacytoid DCs but only mDCs supported viral replication (85)	Human Parainfluenza Virus Type 2 Vector induce DC maturation without viral replication/transcription (86)	Infected DCs can promote airway obstruction, enhance disease, and promote more severe allergic responses A low cDC1:cDC2 ratio correlates with enhanced disease severity (87)	Increase in type I mDCs and a decrease in anti-viral type II mDCs following RV infection in asthmatics (88)
Neutrophils	Significantly fewer neutrophils and inflammatory monocytes were present in the lungs (89)	Significant correlation between MERS-CoV viral load and expression levels of neutrophils chemoattractant chemokines IL-8 (CXCL8) (90)	Activated neutrophils increased (84)	Increased neutrophil influx (91)	Increased neutrophils (92)	Neutrophil chemotaxis and phagocytosis are increased (93, 94)	Not defined
T cells	Lymphopenia (95)	MERS-CoV Efficiently Infects and kill Primary T Lymphocytes (96)	Lymphocytopenia (79) SARS-CoV-2 infects T lymphocytes	High levels of circulating virus-specific CD4^+^ T cells to two viral internal proteins (nucleoprotein and matrix) in the first phase of infection are associated with subsequent development of severe IAV infection (97)	T cells are readily infected by the PIV. The capacity of the virus to regulate T-lymphocyte function may play an important role in the failure of the virus to induce lifelong immunity (98)	Infection with RSV causes a dysregulated antiviral immune response with impaired T cell function as well as exaggerated inflammation via multiple mechanisms (99)	Rhinovirus has the unique ability to bypass antigen presentation and directly infect and activate human T cells (100)
B cells	Lack of peripheral memory B cell responses in recovered patients with SARS (101)	The long-term persistence of antibodies in most patients might be explained by the MERS-CoV infection inducing long-lived memory B cells, which in turn form antibody-secreting plasma cells that are stored in the bone morrow until re-exposure to the same virus or similar epitopes (102)	B cells response against SARS-CoV-2 are detected in the blood around 1 week after the onset of COVID-19 symptoms (103)	Activated B cells differentiate into plasma blasts, the population begins to expand rapidly in the lymph node medulla and secrete predominantly class-switched antibody, peaking between 7 and 14 days post-influenza infection (104, 105)		There is an increase in circulating B cells, including mature (CD19^+^ CD5^+^) and precursor (CD19^+^ CD10^+^) cells, in infants with RSV LRTI, and CD20^+^ B cells and IgM^+^, IgG^+^, and IgA^+^ plasma cells are prominent in postmortem lung tissue from infants with fatal RSV bronchiolitis (106–108)	RVs enter and form viral replication centers in B lymphocytes and induce the proliferation of B cells (109)
Antibodies	Neutralizing antibody responses, likely to the S protein, begin to develop by week 2, and most patients develop neutralizing antibodies by week 3 (110, 111)	The response to MERS-CoV generally occurs through antibody-mediated immunity (112) the neutralizing antibody titers at 34 months of infection in 86% of human serum samples were the same as those after 13 months of infection (113)	Currently, polyclonal antibodies from recovered SARS-CoV-2-infected patients have been used to treat SARS-CoV-2 infection, but no SARS-CoV-2-specific neutralizing mAbs have been reported (114)	Abs elicited against the HA globular domain during infection or vaccination usually are strain-specific, and they will hardly neutralize subsequent influenza virus strains (homosubtypic protection) (115)	Antibodies to the two surface glycoproteins, F and HN are neutralizing and serum and nasal antibody to either protein protects against PIV infection and ameliorates disease (32, 116)	Maternally derived RSV neutralizing antibodies protect infants against RSV hospitalization, and when the infant has recurrent wheeze. However, high maternally derived RSV neutralizing antibody levels were associated with an increased risk of recurrent wheeze (117)	After an RV infection, serum neutralizing antibody titers increase for about a year and high preexisting neutralizing antibody titers have been associated with resistance to reinfection (118)
Cytokines	IFN- γ, IL-10, IL-1β, IL-6, and IL-12 increases IL-4 decreases IL-2 levels increased, while others argued that it decreased (95, 119–121)	MCP-1, MIP-1α and IL-8 chemokines and the cytokine IL-12 are expressed higher in MERS-CoV infection compared to SARS-CoV infection (83, 122, 123) MERS-CoV induced higher levels of IFN-γ, IP-10, IL-12, and RANTES than SARS-CoV (83)	IL-1, IL-6, L-2, IL-7, IL-10, G-CSF, IP-10, MCP-1, MIP-1α, and TNFα increased (124, 125)	IL-6 and chemokines CCL-2/MCP-1, CCL-4/MIP-1β, CXCL-8/IL-8, CXCL-9/MIG, and CXCL-10/IP-10 are associated with pathogenicity of both avian (H5N1 and H7N9) and human (pdmH1N1 and H3N2) viruses. Chemokines CCL-2/MCP-1, CXCL-8/IL-8, CXCL-9/MIG, and CXCL-10/IP-10 are also related with mortality (126)	PIV serotypes differ in their kinetics of replication and cytokine secretion in human trachea-bronchial airway epithelium. PIV1 replicated to high titer yet did not induce cytokine secretion until late in infection, while PIV2 replicated less efficiently but induced an early cytokine peak. PIV3 replicated to high titer but induced a slower rise in cytokine secretion. The T cell chemoattractants CXCL10 and CXCL11 were the most abundant chemokines induced (127)	IL-1, IL-6, IL-10, and CCL5 are increased, while IL-10 and IFN-γ are decreased (124)	Different RV strains can induce different patterns of cytokines and chemokines (128)
Vaccine candidates	No FDA approved vaccine (129)	No vaccine (130)	No vaccine is currently available (131)	Inactivated Influenza Vaccines (IIVs), Recombinant Influenza Vaccine (RIV4) and Live Attenuated Influenza Vaccine (LAIV4) (132)	No licensed vaccine (25)	No vaccine but Palivizumab is a monoclonal antibody recommended to be administered to high-risk infants and young children. It is given in monthly intramuscular injections during the RSV season (54)	No clinically effective rhinovirus vaccine (133)
Treatment	There is no clear, unified and effective treatment plan for COVID-19 (129)	No specific antiviral treatment (130)	Supportive treatment. No specific antiviral drugs (134)	Antiviral drugs may be a treatment option (135)	No antiviral agents symptomatic treatment (32)	Supportive care (136)	There are no approved antiviral medications (137)

ACE2, Angiotensin-converting enzyme 2; SARS-CoV, Severe acute respiratory syndrome coronavirus; SARS-CoV-2, Severe acute respiratory syndrome coronavirus 2; MERS-CoV, Middle East respiratory syndrome coronavirus; DPP4, Dipeptidylpeptidase 4; CX3CR1, CX3C chemokine receptor 1; CDHR3, cadherin-related family member 3; ICAM-1, intercellular adhesion molecule 1; LDLR, low-density lipoprotein receptor; cDC1 and cDC2, conventional Dendritic cells subtypes; AMs, alveolar macrophages; LRI, Lower respiratory infection; sRIRs, Summary relative illness ratios; NFKBIA, Nuclear Factor-Kappa-B Inhibitor Alpha; IFN-γ, Interferon gamma; IL-4, interleukin-4; G-CSF, Granulocyte colony stimulating factor; MIP-la,Macrophage inflammatory protein-1 alpha; MCP-1, Monocyte chemoattractant protein-1; MDMs, monocyte-derived macrophages; MDDCs, dendritic cells; (m)DCs (mDC), type I myeloid. *The basic reproduction number, R0, is the number of secondary infections resulting from a single primary infection into an otherwise susceptible population. It is used to measure the transmission potential of a disease and is the most widely used estimator of how severe an epidemic outbreak can be.

**Figure 1:**
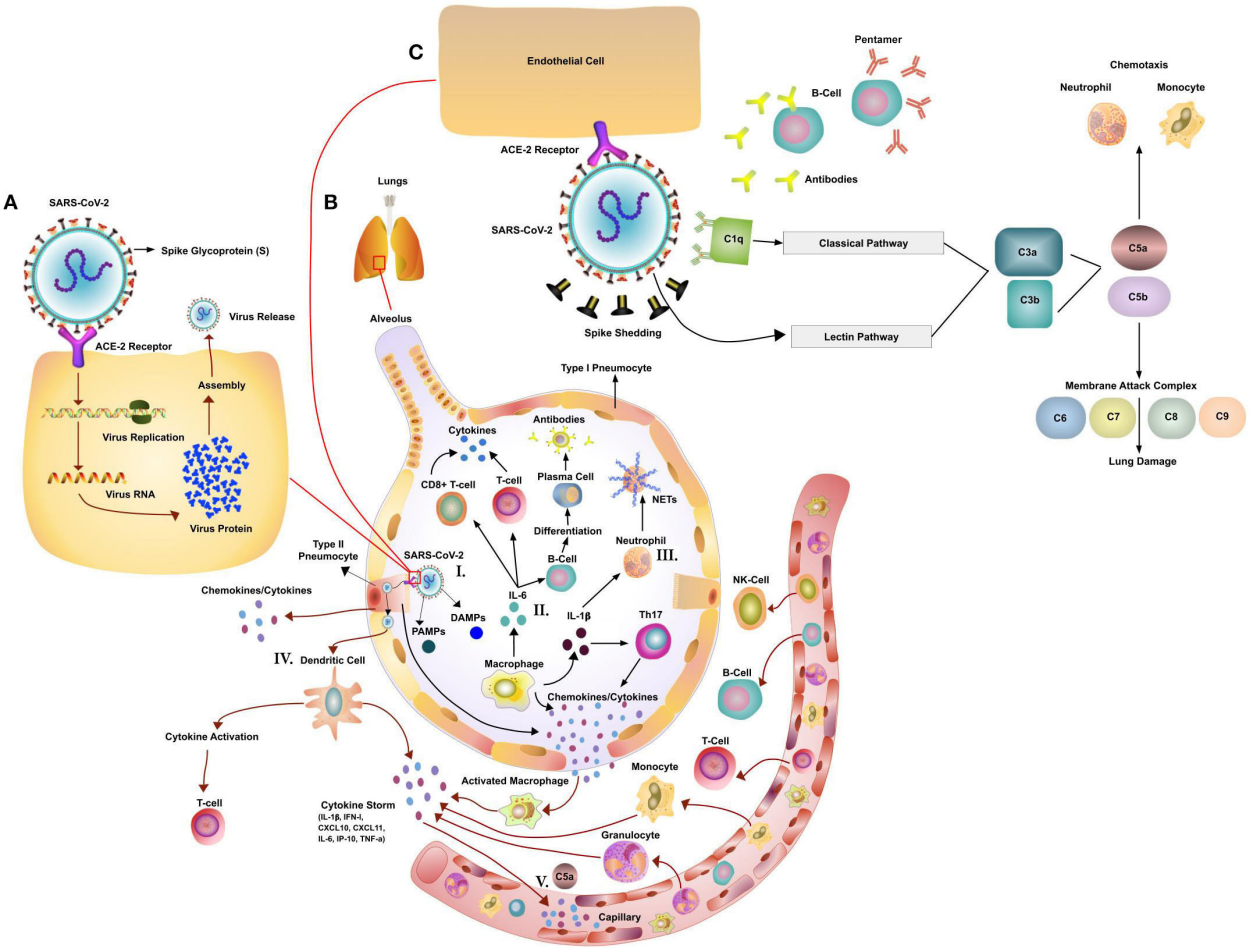
Potential Immune Pathogenesis of SARS-Cov-2. **(A)** Replication cycle of SARS-CoV-2: Spike protein on the SARS-CoV-2 binds to angiotensin converting enzyme 2 (ACE2), a cell-surface protein. The virion releases its RNA. Some RNA is translated into proteins by the host cell's machinery. Proteins and RNA are assembled into a new virion in the Golgi and released. **(B)** The innate and adaptive immune responses to Coronavirus (CoV) infection. (I). Initiation of immune response via PAMPs/DAMPS. The host innate immune system detects CoV infections by using pattern recognition receptors (PRRs) to recognize pathogen-associated molecular patterns (PAMPs) and Damage (Danger)-Associated Molecular Patterns (DAMPs). (II) Activation of T-cells and B-cells via cytokines and activation of the complement system. CoV infection leads to macrophages activation and release of inflammatory cytokines. This in turn activates T and B cells and promotes differentiation. Multiple different T cell subsets (i.e., Th1 and Th17) are involved, releasing cytokines for immune response amplification. (III) Activation of Neutrophils (NET formation) Neutrophils, attracted by chemokines/cytokines swarm to the site of infection. Subsequently activated neutrophils undergo degranulation and NET formation releasing intracellular DAMPs, DNA, histones, neutrophil elastase that activate the PRRs of surrounding immune and non-immune cells to induce cytokine secretion. Neutrophils and neutrophil extracellular traps (NETs) drive necroinflammation in COVID-19. The extracellular DNA released by NETs activates platelets and aggregated NETs provide a scaffold for binding of erythrocytes and activated platelets that promote thrombus formation. (IV) Dendritic Cell mediated activation of T-cells. DCs present viral antigens to T-cells inducing activation. (V) Cytokine and C5a led to influx of immune cells. Secrete chemokines, cytokines and complement C5a attract immune cells. **(C)** Effects of CoV-mediated complement activation. SARS-CoV-2 has been shown to activate the lectin (MBL) complement pathway. Antibodies (early stage IgM or at a later stage IgG) to the virus can activate the classic complement pathway. Both pathways converge at C3. C3 can be converted into C3a and C3b. C3b mediates pathogen opsonization and activates the conversion of C5 into C5a and C5b. C5b mediates the formation of the membrane attack complex, which leads to cell lysis. C3a and C5a promote immune cell recruitment to the site of infection.

While SARS-CoV-2 is a novel coronavirus, several important insights have already been made about its basic mode of transmission. Virus particles are inhaled in respiratory droplets expelled from the airways of infected individuals. Angiotensin-converting enzyme 2 (ACE2), expressed on the ciliated bronchial cells, endothelial cells, and type I and II alveolar cells, is the host receptor for cell entry into the respiratory tract by both SARS-CoV-2 and SARS-CoV (Table 1) (147–150). The spike protein (S) of CoV is responsible for the entry of the virus into the target cell (Figure 1) (147, 151). ACE2 is a type I transmembrane metallocarboxypeptidase that plays a vital role in the Renin-Angiotensin System (RAS) (152, 153), which in turn is critical in maintaining blood pressure homeostasis as well as fluid and salt balance in mammals. ACE2 is found in vascular endothelial cells, in the renal tubular epithelium, and in Leydig cells of the testes (154). Studies have shown that ACE2 is expressed in gastrointestinal (GI) tissues, making it a potential site for harboring SARS-CoV (155). This may be one of the reasons for GI pathology reported in some patients with COVID-19 and viral shedding in stool. In contrast, MERS-CoV uses dipeptidyl-peptidase 4 (DPP4) as an entry receptor, which is expressed on endothelial cells and some epithelial tissues (Table 1) (19, 156).

Accumulating data suggest that the innate anti-viral response and adaptive immunity may contribute to a cytokine storm leading to systemic hyper inflammation and exacerbation of the disease in patients with (a) co-morbidities (b) older than 65 years of age (c) of the male sex. The exact role of the innate immune system in disease pathogenesis and prevention between the sexes and the impact of age is not fully elucidated. In addition, the potential dysregulation of the innate immune response by SARS-CoV and SARS-CoV-2 is not completely understood which warrants further research.

## Respiratory Epithelial Cell and Resident Innate Immune Cell Responses to Respiratory Viral Infections

The cells of the airway epithelium are the first line of defense, providing a mechanical barrier (mucociliary escalator) that expels particles and pathogens via cilia, mucus, and induced coughing (157, 158). This barrier includes cells of the pulmonary epithelium and tissue-resident macrophages and dendritic cells (DCs). The macrophages and DCs express pattern recognition receptors (PRRs) that can detect molecules from pathogens (Pathogen-Associated Molecular Patterns—PAMPs) or molecules released from damaged cells (Damage or Danger-Associated Molecular Patterns—DAMPs) (158–160). In the lung, there are two populations of macrophages, alveolar and interstitial macrophages (161). In addition to these macrophages, DCs play a vital role in facilitating the host defenses against respiratory diseases (162–164). DCs can be divided into plasmacytoid (pDC) and myeloid types (mDC) (165–167). Macrophages and the two DC subtypes trigger antiviral responses by generating a substantial amount of type I interferon and these cells play important roles in immune surveillance in the airways and the distal lung (74, 168–172). During steady state, the DC density in lung associated tissue declines from the trachea to the alveoli (173) while the representation of cells in macrophage compartment seems more complex (174). If a virus infects airway epithelial cells, the viral RNA would be sensed via intrinsic innate receptors, including RIG-1, MDA5, NLRP3 inflammasome, and the RNA sensing TLRs 7 and 8. In the case of influenza A infection, triggering the PRRs causes a strong induction of type 1 interferon (IFN) in epithelial cells (175, 176). In other viral infections, such as Respiratory Syncytial Virus (RSV), alveolar macrophages are the predominant source of type 1 IFNs (161). Furthermore, respiratory epithelial cells and lung macrophages are capable of secreting a broad range of chemokines like IL-8, Macrophage inflammatory protein-1 (MIP-1), RANTES and cytokines including TNF-α, IL-6, IL-1β that influence the types of immune cells being recruited to the area in response to acute viral infections (177, 178).

Macrophages, depending on their polarization status of either M1 or M2, and in a similar way as airway epithelial cells, can further elicit a Th1 or Th2 response (158, 161, 178). In the case of influenza virus infection, the magnitude of epithelial cell response can be proportional to the amount of virus which result in paracrine induction of IFN λ (175). Not only can airway epithelial cells produce a large array of cytokines/chemokines in response to an acute viral infection, but, depending on the magnitude of PRR engagement and the combination of PAMPs and DAMPs triggered, these epithelial cells can modulate the type of chemokines/cytokines produced and influence the influx of innate and adaptive immune cells (158, 160). The response to different viral infections is generally similar, however, the response can be tailored in timing, magnitude and the induced gene signatures in response to each virus (179). Unlike RSV and MERS-CoV, which both productively infect alveolar macrophages (73, 180), seasonal influenza and SARS-CoV usually lead to non-productive infections in these cells (181). In addition, SARS-CoV infection of primary monocytes yielded little virus, likely due to the suppressive effects of IFN-α (182). Thus, the initial cell type(s) involved in propagating a viral infection intensifies the complexity of the immune response.

Another key factor that determines the magnitude of the immune response is the induction and rate of cell death. Although related, MERS-CoV induces widespread cell death when compared to SARS-CoV in human bronchial epithelial cell cultures (183). However, the SARS-CoV open reading frame 3a (ORF3a) protein can induced necrotic cell death in a variety of cell lines (184). The same ORF3a protein can activate the NLRP3 inflammasome, leading to activation of NF-κB and elevated secretion of active IL-1β in cell culture (185).

Cytokine profiles of macrophages activated by SARS-CoV and MERS-CoV are different (180). Nonetheless, both MERS-CoV and SARS-CoV, in human epithelial cell and fibroblast culture, show a delay (24–30 h post-infection) in the induction of proinflammatory cytokines (186), with slightly different cytokine/chemokine profiles. This delay in cytokine induction was confirmed in another study using the same epithelial cell lines (187) as well as in human alveolar type II cells (18). In both cell lines and primary alveolar type II cells, SARS-CoV induced IFN-β, IFN-λ, CXCL10, CXCL11, IL-6, IP-10, and TNF-α (18, 187). MERS-CoV did not induce IFN-β but induced higher level of IL-8 transcript in cell culture. However, no difference in IL-8 production was observed between SARS-CoV and MERS-CoV at 48 h post-infection (186). This was confirmed *in-vivo* using a non-human primate model comparing the immune responses to SARS-CoV infection between young adult cynomolgus macaques (3–5 yrs) and older macaques (10–19 yrs) (188).

Interestingly, the high induction of IL-8 was observed on a transcript level in the older animals, while the younger once showed higher levels of IFN-β transcript (188). In all animals, the expression of IFN-β was inversely correlated with the pathology score, supporting the role of IFN-β in controlling disease severity (188) and introducing a potential area of research to define age disparity observed in patients infected with SARS-CoV-2. Both older age and male sex are important factors associated with high mortality of SARS-CoV and SARS-CoV-2 infection (189, 190). Many viruses have evolved to disrupt and subvert the immune responses. A common virus that is well-known to affect the lower airway and counteract the immune response is RSV (178, 191). The RSV genome encodes non-structural proteins NS1 and NS2 that can block type 1 IFN production and signaling in cell cultures (191). Similar to RSV, the Measle virus V protein blocks IFN-α and β signaling by inhibiting Stat1 and Stat2 signaling in cell lines (192), whereas MERS-CoV M protein also suppresses type 1 IFN by inactivating IRF3 (193), leading to the low expression of IFN-β.

In contrast to reports in epithelial cell lines or primary alveolar type II cell culture and observations in non-human primates, SARS-CoV nucleocapsid (N) protein and membrane (M) protein, as well as nsp1, can suppress IFN response via various mechanisms in cell lines (194–196). To bridge the dichotomy of inhibition of IFN signaling in cell lines, and the IFN expression *in vivo*, cells recruited by the infection need to be considered as a potential source. As previously discussed, infected epithelial cells via paracrine signaling to neighboring cells and resident macrophages, secrete chemokines and cytokines to attract other immune cells.

In general, monocytes/macrophages are recruited by CCL3 (MIP-1a), CCL2 (MCP-1), and neutrophils are recruited by IL-8 (CXCL8), CXCL2, and CXCL5 chemokines, all of which can be secreted by airway epithelial cells (178, 197, 198). Both monocytes and neutrophils are also recruited by complement fragment (anaphylatoxin) C5a (Figure 1). Both Influenza and SARS virus can induce acute lung injury (ALI) which is accompanied by high levels of C5a, leading to the influx and activation of innate immune cells (199) (Figure 1).

Serum samples and lung tissue of SARS patients showed high-level expression of CXCL10 (IP-10), which is also found to be induced by SARS-CoV in the epithelial cell line Calu-3 (200). Significant neutrophil, macrophage and CD8 T-cell infiltration can be found in the lung of SARS patients by immunohistochemistry (76, 77, 201). In addition to post-mortem lung histology analysis in patients with SARS-CoV, experiments using Rhesus macaques infected with SARS-CoV found monocyte and macrophage recruitment. The accumulation of pathogenic inflammatory monocyte-macrophages (IMMs) was also observed in a SARS-CoV mouse model. The accumulation of IMMs resulted in heightened lung inflammatory cytokine/chemokine levels, extensive vascular leakage, and impaired virus-specific T cell responses (202).

A strong infiltration of CD68 and Mac387 positive monocytes/macrophages were found in the human and animals lung samples (203, 204). Macrophages further stimulate fibroblasts to deposit extracellular matrix leading to pulmonary fibrosis (205), which was also observed in patients who recovered from SARS (206, 207). Autopsy samples acquired from patients with SARS-CoV-2 patients contained viral nucleocapsid protein (NP) positive CD68^+^ macrophages in the capillaries of the spleen and in lymph nodes, indicating that SARS-CoV-2 might migrate into the spleen and lymph nodes through macrophages. This study also found that CD169^+^ macrophages appear to mediate SARS-CoV-2 into these tissue sites, contributing to virus dissemination (208). Similar to SARS-CoV-2, SARS viral particles and genomic sequences were detected in monocytes, macrophages as well as within different organs of SARS patients (15). SARS-CoV was shown to infect both immature and mature human monocyte-derived DCs by electron microscopy and immunofluorescence. The detection of negative strands of SARS-CoV RNA in DCs suggests viral replication, but no increase in viral RNA was observed (209). As mentioned above, there was no perceivable increase to SARS-CoV replication in primary monocytes (182). Another study looked at SARS-CoV and MERS-CoV replication in human macrophages, human monocyte-derived macrophages, and dendritic cells (MDDCs) and found that both viruses replicated poorly. MERS-CoV induced IFN-λ1, CXCL10, and MxA mRNAs in both macrophages and MDDCs, however, SARS-CoV was unable to induce such responses (69). Interestingly, depletion of inflammatory monocyte-macrophages in the mouse model partially protected from lethal SARS infection (210). These data suggest that monocytes, macrophages and dendritic cells have essential roles in CoV infection. The severity of disease and the response to these viruses seems to be dependent on the induced cytokine/chemokine profiles and the amplification of the immune response by the recruited cells. Growing evidence of dysregulation of an innate anti-viral response originates from studies using clinical samples (211) and murine models (202, 212, 213).

In addition to dysregulated cellular responses, the complement system may play an important role in SARS-CoV-2 infection (Figure 1). Evidence comes from SARS infected patients who had lower levels of mannan binding lectin (MBL) in serum compared to healthy controls (214). The SARS patients with a higher frequency of MBL gene polymorphisms were associated with lower serum levels or deficiency of MBL (214). It is still unknown if this is also true for COVID-19 patients, which requires further investigation. In cell culture experiments SARS-CoV was able to bind and activate the complement cascade and block viral infection (214). Preliminary findings in a limited number COVID-19 patients found significant deposits of the membrane attack complex (MAC), C4d and MBL-associated serine protease (MASP)2 in the microvasculature indicating sustained, systemic activation (215). The SARS-CoV-2 spike protein was co-localized with C4d and MAC (215). In a non-peer reviewed publication by Gao et al., MERS-CoV, SARS-CoV and SARS-CoV-2 N protein are able to bind to MASP-2 leading to enhanced complement activation (216) (Figure 1). In a later phase of the infection, the complement system might be also triggered via antibodies bound to the virus (Classic activation pathway, Figure 1). This excessive complement activation might play a role in multi organ damage in severe COVID-19 cases (217). In a MERS-CoV mouse model the blockade of the C5a-C5aR axis alleviated not only lung damage but also spleen damage (218). Mice treated with a monoclonal antibody to C5a showed reduced lung infiltration of CD68^+^ cells and significantly lower cytokine levels of IL-1 β, TNF-α, INF-γ and IL-12 (218). Complement blockade might be an important way to curb part of the immune dysregulation associated with COVID-19. Overall, we need to look closer at the role of the complement system, the recruited innate immune cells and their combined role in pathogenesis, viral clearance and the eventual resolution of the infection.

## The Role of Neutrophils

The most abundant leukocytes, neutrophils, play a critical role in clearing viral infections. Neutrophils, attracted by chemokines/cytokines released by tissue-resident macrophages and DCs, swarm to the site of infection. They recognize and release bioactive compounds, including cytokines, chemokines and ROS, as well as NOS in the very early phase of the infection (219, 220). Neutrophils modulate other innate and adaptive immune responses via cytokine/chemokine release and cell death and, therefore, can ameliorate or exacerbate disease progression. Neutrophils infiltrate tissues infected by CoV, including SARS-CoV, Rat coronavirus (rCoV), and Mouse Hepatitis Virus (MHV). A high neutrophil count in the blood of SARS patients at the time of hospital admission is associated with a poor prognosis (221, 222). Gao et al. suggested that patients with SARS-CoV-2 pneumonia can be stratified by neutrophil to lymphocyte ratio (NLR) and age (216). Patients older than 50 years of age and having an NLR ≥ 3.13 had more severe illness, so rapid access to the intensive care unit is required (79, 223). Experiments in mice showed that SARS-CoV disease severity in older mice correlated with increased pulmonary inflammation and influx of neutrophils (224, 225). Infection of rats with rCoV could lead to neutrophil infiltrating into the respiratory tract early after inoculation, followed by the recruitment of macrophages and lymphocytes (226). Infection of mice with a neurotropic murine CoV (MHV-JHM) showed infiltration of neutrophils into the brain as early as the first day after inoculation, which then promoted the recruitment of other types of inflammatory cells into the brain, likely through the loss of the blood-brain barrier integrity (227). Gene expression analysis in experimentally infected rhesus macaques with MERS-CoV revealed the recruitment of neutrophils into infected lung tissue (228, 229).

Angiotensin-converting enzyme inhibitors (ACE-Is) could serve as a potential risk for fatal COVID-19 through the up-regulation of ACE2 (230) and may provide a direct linkage to neutrophils and disease progression. Investigators found that ACE2 modulates IL-17-mediated neutrophil influx by impacting STAT3 activity (231). Animal models used to study the pathogenesis of SARS-CoV-2 have revealed important roles of neutrophils in infection and confirmed findings observed in patients. A new aspect in SARS-CoV-2 infection is the potential role of neutrophil extracellular traps (NETs). The process of NET formation is a specific type of cell death that can be triggered under inflammatory conditions (232, 233), such as GM-CSF+C5a, IL-8, IFN-α+C5a or other TLR response mediators; all conditions present in severe SARS-CoV-2 infection (232, 233). The NET formation has been observed in COVID-19 patients and may contribute to thrombotic complications in COVID-19 patients (234, 235). Microvascular injury and thrombosis have been reported in COVID-19 patients, increasing the likelihood that neutrophil NET formation might play a role (215, 236, 237). NET formation was reported to be involved in clot formation and thrombosis and can further increase inflammation (232, 233). Therefore, neutrophils can attract a second wave of immune cells to the site of infection by cytokine/chemokine secretion as well as via NETosis (238, 239), which included monocytes and natural killer cells. On the other hand aggregated NETs were reported to limit inflammation by degrading cytokines and chemokines and disrupting neutrophil recruitment and activation (240). Despite the presence of neutrophils in SARS-CoV-2-infected tissues, their role in the clearance and/or immunopathology of the viral infection remains unclear. Future studies on the responses of neutrophils to SARS-CoV-2-infection or infected cells *in vitro* may elucidate the role of neutrophils in the pathogenesis of respiratory CoV infections.

## The Potential Role of Natural Killer Cells

Natural Killer (NK) cells are a heterogenic immune cell subset that acts promptly to combat viral infections. They produce significant amounts of IFN-γ, kill virus-infected cells, provide direct support to other innate immune cells, and aid in the adaptive immune response to counter viruses. NK cells express activating receptors that detect viral antigens, enabling the destruction of infected cells (241–244). Lectin-like receptor CD94 and killer immunoglobulin-like receptors, such as CD158b, regulate the function of NK cells. A study of 221 patients with SARS explored the relationship of the number of NK cells and the expression level of their immunoglobulin-like receptor CD158b in the peripheral blood to the severity of SARS (245). The overall count of NK cells and CD158^+^ NK cells and the percentage of CD158^+^ NK cells in patients with SARS were significantly lower than counts in healthy subjects (245). A separate study that analyzed lymphocytes and lymphocyte subsets in a cohort of 38 patients with SARS observed reduced NK cell counts in 21 patients (55%) (246). Clinical reports reveal that children appear to have a milder form of SARS-CoV-2, with peripheral blood lymphocyte levels remaining in the normal range, suggesting less immune dysfunction following the disease (247). This could be attributed to healthy children expressing lymphocytes, especially NK cells, in a greater quantity compared to healthy adults (248). Interestingly, previous studies found rapid and significant restoration of lymphocyte subsets including, NK cells, in peripheral blood in patients recovering from the initial stages of SARS infection (249). Although the primary mechanism for the decrease in NK cells and other subsets during disease onset remains unknown, their contribution to SARS-CoV-2 needs further study especially from a treatment perspective.

## The Contribution by the Innate Lymphocytes

Innate lymphoid cells (ILCs) are a family of innate immune cells that include ILC1, ILC2 and ILC3. Although ILC2 facilitates lung repair after injury, the role of ILCs during respiratory viral infection is not clearly defined (250). Evidence for the potential involvement of ILC2 cells in the lung during viral infection was reported in a murine model (251). This study found a rapid accumulation of ILC2 cells in the lung after an influenza virus infection, however their initial contribution to exacerbation of the disease was limited (251). A recent study identified an interaction between ACE2-expressing SARS-CoV-2 target cells and ILCs in the colon (252). Thus, elucidating the role of ILC subsets will be important in understanding the pathogenesis of SARS, SARS-CoV-2 and MERS infections.

## Roles of Interferons in Coronavirus Infection

There is distinct evidence indicating an important role of IFNs in SARS and other CoV infections (201, 253). The sera of patients with SARS revealed the presence of high levels of IL-1, IL-6, INFγ, CCL2, CXCL10, and IL-8 and products of interferon stimulated genes (254, 255). High expression levels of ISGs such as CD58, IFNAR1, and IFNGR1 and IFN-stimulated chemokines CXCL10 and CCL2 were observed in another cohort of SARS patients and were correlated with the severity of pathogenesis (256). Significant upregulation of CXCL10 gene expression was observed in the severe phase of patients who died from SARS. This data is corroborated by studies in patients with MERS that found upregulation of CXCL10 in the serum of patients who developed pneumonia (254). CXCL10 and INFα were also correlated with severity of disease (255).

The importance of IFN signaling in response to CoV infection has been well-demonstrated in several knockout mouse models. Type I, II, and III IFN signaling deficient mice have increased susceptibility to mouse-adapted SARS-CoV strains (257, 258). Studies using mice lacking the IFNAR1 and IFNLR1 or STAT1 identified higher SARS-CoV replication in the lungs and delayed virus clearance (259, 260). Another study with MERS-CoV in mice expressing the human DPP4 receptor showed a role for the IFNAR1 in viral clearance and lung inflammation (112). These mouse models suggest an important role of IFN response for CoV clearance. This quick expanding medical literature is very suggestive of an important role of IFN responses for CoV control and clearance.

## Innate Immune Evasion Strategy of Human Coronaviruses

Many viruses have evolved to disrupt and subvert the immune response. RSV counteracts the immune response (178, 191); as discussed earlier, the RSV genome encodes non-structural proteins (NS1 and NS2) that are able to block type 1 IFN production and signaling in cell cultures (191). Similar to RSV, the Measle virus V protein blocks IFN-α and β signaling by inhibiting Stat1 and Stat2 signaling in cell culture lines (192). CoVs have developed several ways to escape from innate immune pressure. MERS-CoV M protein suppresses type 1 IFN by blocking the IRF3 activation (193), explaining the low expression of IFN-β. In various cell lines, SARS-CoV nucleocapsid (N) protein, membrane (M) protein, as well as nsp1, were reported to suppress IFN response (194, 196, 261). The nucleocapsid protein (N) of SARS-CoV interferes with the function of IRF3. Although it does not form a complex with RIG-I or MDA5, RNA binding activity at the initial recognition stage of viral RNA potentially contributes to immune evasion (261, 262).

Aside from the HCoV, structural proteins, accessory, and non-structural proteins (nsp) are involved in innate immunity modulation. In both SARS-CoV and MERS-CoV, host mRNA endonucleolytic cleavage is promoted by nsp1 protein, which modifies capped non-viral RNAs (263, 264). Nsp1 in SARS-CoV prevents host mRNA translation by interacting with the 40S subunit of the ribosome; in turn, transcription and translation of viral RNA is given preference over the host mRNA (263). Another study found that additional SARS-CoV nsp1 residues interfered with IFN-dependent signaling (265). IFN production is affected by nsp3 proteins in SARS-CoV and MERS-CoV. These proteins have both papain-like protease (PLpro) and a PLP2 domain, and the PLpro domains in both SARS-CoV and MERS-CoV downregulate mRNA levels of CCL5, INFβ, CXCL10, and other pro-inflammatory cytokines (266). The suppression of IFN responses by SARS-CoV PLpro is due to the inhibition of phosphorylation of IFN-regulatory factor 3 (IRF3) and its subsequent translocation to the nucleus where it enhances IFN gene transcription (267). MERS-CoV PLpro also suppresses RIG-I and MDA5 and antagonizes IFN induction (266, 268). In HCoV-229E and SARS-CoV suppression of IFN responses, the key molecule is a ADP-ribose-1-monophosphatase macrodomain encoded within nsp3 (269). Accessory proteins are not key in viral replication; however, in human CoV, this group of proteins are involved in diverse cellular signaling, including cell proliferation, apoptosis, and IFN signaling (270). By downregulating phosphorylation and nuclear translocation of IRF3, Open Reading Frame ORF3b and -6 interfere with IFNβ synthesis and prevent IFNβ-induced activation of IFN-stimulated response element (ISRE) in the promoter of ISG in SARS-CoV (262). In cells transfected with ORF4a, -4b, and -5 of MERS-CoV, IFNβ promoter-driven luciferase activity is significantly reduced, and it may follow a similar pattern of suppression of IRF3 nuclear translocation (141). Therefore, the suppression of signaling events in infected immune and airway epithelial cells, as well as the magnitude of suppression due to elevated expression levels of these accessory proteins, needs to be further elucidated to understand delayed or hyperimmune responses and cytokine storm that occurs in CoV infection.

## Summary

In addition to revealing our unpreparedness of handling a worldwide pandemic by a viral infection, COVID-19 exposed our lack of understanding of the pathogenesis of diseases as well as the host immunity. The interaction of the host innate immune system and other factors including age, sex, and pre-existing conditions need further investigation regarding disease severity and morbidity of SARS/MERS and COVID-19. Disease severity and its related progression are further associated with dysregulation of multiple components of both innate and adaptive immune responses leading to a cytokine storm and severe pathology. For the development of a therapeutic intervention, it is vital to elucidate the interplay among the different layers of the innate immune response and their relation to the clinical factors associated with increased morbidity and mortality in CoV infection. Investments in basic science research are needed to help elucidate the roles of different immune cells, and their contribution to disease severity; it will pave the way to prevent or abrogate CoV outbreaks and potentially new viruses.

## Author Contributions

NL, TD, DH, ZL, and NF performed the literature search, analyzed the literature, and wrote the manuscript. HA-H, BS, YW, and RS performed the literature search and wrote the manuscript. All authors contributed to the article and approved the submitted version.

## Conflict of Interest

TD is employed by company Marker Therapeutics Inc. The remaining authors declare that the research was conducted in the absence of any commercial or financial relationships that could be construed as a potential conflict of interest.
